# Selective screening for thyroid dysfunction in pregnant women: How often do low-risk women cease to be treated following the new guidelines of the American Thyroid Association?

**DOI:** 10.20945/2359-3997000000089

**Published:** 2018-10-01

**Authors:** Pedro Weslley Rosario

**Affiliations:** 1 Santa Casa de Belo Horizonte Belo Horizonte MG Brasil Santa Casa de Belo Horizonte, Belo Horizonte, MG, Brasil

**Keywords:** Screening, thyroid dysfunction, pregnancy

## Abstract

**Objective:**

Universal screening for thyroid dysfunction in pregnant women is not recommended by the American Thyroid Association (ATA) or the American Association of Clinical Endocrinologists (AACE). This study evaluated the frequency of pregnant women that would have an indication for levothyroxine (L-T4) according to the new ATA/AACE guidelines among low-risk women without an indication for screening with TSH.

**Subjects and methods:**

The sample consisted of 412 pregnant women ranging in age from 18 to 30 years. These women were considered to be at low risk for thyroid dysfunction according to ATA/AACE and would not be candidates for screening with TSH. Anti-thyroid peroxidase antibodies (TPOAb) and TSH were measured. Women who had TSH > 2.5 mIU/L or TPOAb in the first trimester were submitted to subsequent evaluations in the second and third trimester.

**Results:**

In the first trimester, none of the pregnant women would have L-T4 therapy “recommended” and treatment would be “considered” in only two. In the second trimester, pregnant women with positive TPOAb or TSH > 2.5 mIU/L in the first trimester (n = 30) were reevaluated. L-T4 treatment would be “recommended” in only one woman and would be “considered” in two others. The 28 women that were not treated in the second trimester were reevaluated in the third trimester, but none of them would have L-T4 “recommended”.

**Conclusion:**

The findings of the study suggest that selective screening, recommended by ATA/AACE does not result in a significant loss of pregnant women with an indication for L-T4 treatment.

## INTRODUCTION

The most recent guidelines of the American Thyroid Association (ATA), reviewed and endorsed by the American Association of Clinical Endocrinologists (AACE), recommend that: (i) when indicated, screening for thyroid dysfunction in pregnancy should be performed during the first prenatal visit, which generally occurs in the first trimester; (ii) this screening starts with the measurement of serum TSH, and (iii) TSH between 0.1 and 2.5 mIU/L closes the investigation (1). Universal screening for thyroid dysfunction in pregnant women is not recommended by ATA/AACE (1). The recommendation is that only high-risk pregnant women be investigated by TSH measurement (1). The definition of this group was also reinforced in the recent guidelines (1). Although several conditions are considered risk conditions, many argue that the selective screening strategy (1) fails to diagnose thyroid dysfunction in a significant portion of low-risk pregnant women, thus recommending universal screening (2,3).

Controversy regarding screening for thyroid dysfunction in pregnant women also exists in Brazil. In the consensus on “the clinical use of thyroid function tests”, in addition to individuals with risk factors, pregnancy is an indication for TSH measurement (4). On the other hand, the consensus on subclinical hypothyroidism concluded that “there is insufficient evidence to recommend or not recommend universal screening for hypothyroidism with TSH in pregnant women in the first trimester of gestation” (5). This divergence is also observed in clinical practice. A Latin American study showed that 38.4% of responders use a universal screening strategy and 43% prefer a case-finding approach in high-risk groups (6). Finally, the recommendations for levothyroxine (L-T4) treatment were revised in the recent ATA/AACE guidelines (1).

To our knowledge, no Brazilian study has evaluated the frequency of pregnant women that would have an indication for L-T4 treatment according to the new ATA/AACE guidelines (1) among women with low risk and therefore without an indication for screening with TSH (1). This was the objective of the present study.

## SUBJETCS AND METHODS

The study was approved by the local Research Ethics Committee (7,8). The population studied was from the metropolitan region of Belo Horizonte (Minas Gerais, Brazil), an area where iodine intake is adequate. Nine hundred and ninety-two pregnant women with ≤ 12 weeks gestation who underwent prenatal tests at a clinical analysis laboratory and who had become pregnant spontaneously were initially interviewed and examined (7,8). For this study, women who met the clinical criteria shown in Table 1 (n = 480) were excluded. The sample consisted of 412 women ranging in age from 18 to 30 years, with a median gestation of 9 weeks, including 212 primigravidae. These women were at low risk for thyroid dysfunction according to ATA/AACE and would not be candidates for screening with TSH (1).

**Table 1:** Exclusion criteria

Known thyroid disease, current or previous treatment with antithyroid drugs or L-T4
History of ^131^I therapy, thyroidectomy or head and neck external radiotherapy
Age > 30 years
Type 1 diabetes or other autoimmune diseases
History of pregnancy loss, preterm delivery, or infertility
Multiple prior pregnancies (≥ 2)
Family history of autoimmune thyroid disease or thyroid dysfunction
Morbid obesity (BMI ≥ 40 kg/m^2^)
Use of amiodarone, interferon, or lithium; or recent (in the past 8 weeks) exposure to iodinated contrast agents
Goiter, palpable thyroid anomaly or ophthalmopathy

Anti-thyroid peroxidase antibodies (TPOAb) and TSH were measured. Only women who had TSH > 2.5 mIU/L or positive TPOAb in the first trimester were submitted to subsequent evaluations in the second and third trimester.

Regarding L-T4 therapy, two categories were defined according to the ATA/AACE guidelines (1): (i) therapy “recommended”, if TSH > 10 mIU/L or TSH entre 4 and 10 mIU/L with positive TPOAb, and (ii) therapy “considered”, if TSH between 4 and 10 mIU/L without TPOAb or TSH between 2.5 and 4 mIU/L with positive TPOAb.

Serum samples were obtained from the women in the morning (at about 8 a.m.) after an 8- to 10-h fast. TSH was measured with a chemiluminescent assay (Immulite 2000, Diagnostic Products Corporation, Los Angeles, CA), with reference values of 0.4-4 mIU/L. TPOAb were also measured with a chemiluminescent assay (Immulite 2000), with reference values of up to 35 kIU/L.

## RESULTS

In the first assessment (first trimester), none of the pregnant women would have L-T4 therapy “recommended” and treatment would be “considered” in only two. These women had positive TPOAb and TSH between 2.82 and 3.12 mIU/L. None of the women was treated with L-T4 in this first assessment. Of note, none of the women would be a candidate for antithyroid drug treatment [TSH < 0.1 mIU/L with elevated free T4 and positive anti-TSH receptor antibodies (TRAb) (1)].

Pregnant women with positive TPOAb (n = 10) or TSH > 2.5 mIU/L (n = 18) or both (n = 2) in the first assessment were reevaluated in the 22^nd^ week of gestation (second trimester). L-T4 treatment would be “recommended” in only one woman (she had TSH 4.8 mIU/L and positive TPOAb) who was actually treated. In other two patients, L-T4 therapy would be “considered”, one with TSH 3.5 mIU/L and positive TPOAb and the other with TSH 4.5 mIU/L without TPOAb. L-T4 was initiated only in the first patient.

The 28 women who were not treated in the second trimester were reevaluated in the 34^th^ week of gestation (third trimester), but none of them would have L-T4 treatment “recommended” (1). The woman for whom therapy would be “considered” in the second trimester (1) continued with this classification in the third trimester.

## DISCUSSION

Considering that pregnant women with TSH < 2.5 mIU/L without TPOAb would remain without an indication for L-T4 therapy throughout pregnancy (1) and applying the recommendations of the new ATA/AACE guidelines (1), among the 412 women at low risk for thyroid dysfunction (1), only one would have treatment “recommended” and treatment would be “considered” in two (1).

Population differences may exist, and it is possible that our results cannot be reproduced in all populations. Furthermore, pregnant women with TSH < 2.5 mIU/L without TPOAb were not reevaluated in the second or third trimester, but these women do not require additional investigation according to ATA/AACE (1). In addition, since these women did not have TPOAb, L-T4 treatment would only be “considered” if TSH exceeds 4 mIU/L and only above 10 mIU/L would treatment be “recommended” (1), which we believe is highly unlikely to occur. Despite these limitations, the study included a reasonable number of pregnant women who were rigorously selected and considered to be at low risk for thyroid dysfunction following the definition of ATA/AACE (1). In addition, the treatment indications were evaluated according to the most recent guidelines of ATA/AACE (1).

The findings of the study suggest that selective screening, recommended by ATA/AACE (1), does not result in a significant loss of pregnant women with an indication for L-T4 treatment. Since in the present series low-risk pregnant women accounted for less than half of the initial sample, the high-risk definition proposed by ATA/AACE (1) seems to be poorly selective but has a high negative predictive value.
